# Exploration of singular and synergistic effect of xylitol and erythritol on causative agents of dental caries

**DOI:** 10.1038/s41598-020-63153-x

**Published:** 2020-04-14

**Authors:** Siiri Kõljalg, Imbi Smidt, Anirikh Chakrabarti, Douwina Bosscher, Reet Mändar

**Affiliations:** 1 Department of Microbiology, Institute of Biomedicine and Translational Medicine, University of Tartu, Tartu, Estonia; 2 Cargill R&D Centre Europe, Vilvoorde, Belgium

**Keywords:** Pathogens, Biofilms, Oral diseases, Infection

## Abstract

Non-cariogenic sweet substances, like sugar alcohols, are used to decrease the risk of caries by reducing the growth of dental plaque. The aim of our study was to reveal the impact of xylitol and erythritol on the growth and biofilm formation of cariogenic bacteria including as a novelty, set of clinical mutans streptococci and *Scardovia wiggsiae* and to assess the possible synergistic influence of these polyols. We found both xylitol and erythritol to express high growth inhibition effect on cariogenic bacteria. In synergistic effect experiments, 10% polyol combination with excess of erythritol was found to be more effective against growth of *Streptococcus mutans* and the combination with excess of xylitol more effective against growth of *Streptococcus sobrinus* and *S. wiggsiae*. In biofilm inhibition experiments, solutions of 10% polyols in different combinations and 15% single polyols were equally effective against mutans streptococci. At the same time, higher biofilm formation of *S. wiggsiae* compared to experiments without polyols was detected in different polyol concentrations for up to 34%. In conclusion, both erythritol and xylitol as well as their combinations inhibit the growth of different cariogenic bacteria. Biofilm formation of mutans streptococci is also strongly inhibited. When applying polyols in caries prophylaxis, it is relevant to consider that the profile of pathogens in a particular patient may influence the effect of polyols used.

## Introduction

Contemporary theories support the role of non-cariogenic sweet substances, like sugar alcohols, as the one way to reduce the risk of caries. Clinical investigations have shown that xylitol (XYL), a pentitol type sugar alcohol, can be used as a safe and effective caries-limiting sweetener^1^. Regular use of oral hygiene adjuvants and xylitol-containing products has shown to inhibit the growth of caries-associated bacteria, to cut the growth of dental plaque and to decrease the incidence of dental caries^1^. There are numerous science-based regulatory propositions for the use of xylitol as a caries-limiting agent^2^.

Recently, a tetritol-type alditol, erythritol (ERY), has shown potential as a non-cariogenic sugar substitute. Erythritol is well tolerated in the gastrointestinal tract and well absorbed in small intestine but not metabolized in the body and thereby it is a non-caloric polyol. Erythritol has been shown to significantly and in larger extent than xylitol to reduce the dental plaque weight^3,4^. Erythritol’s mechanism of action is generally similar to xylitol and it is to reduce growth of the plaque-related biofilm, and streptococci do not produce neither lactic nor other acids from erythritol^2,4–7^.

Etiology of caries in humans is mostly associated with mutans streptococci, namely *Streptococcus mutans* and *Streptococcus sobrinus*^8^. *In vitro* experiments have shown direct inhibitory effect of erythritol and xylitol on the growth of mutans streptococci^3,9^. It has been supposed that combinations of xylitol and erythritol may reduce the incidence of caries more effectively than either alditol alone^2^, however, synergistic inhibitory effect of xylitol and erythritol on mutans streptococci needs to be confirmed first.

Additionally, the influence of polyols on etiological factors of caries has mainly been focused on mutans streptococci and no information about polyol effect on newly discovered oral pathogen associated with childhood caries, *Scardovia wiggsiae*^10^ is available so far. As mostly only type strains are used in experiments studying polyol efficacy the information about number of clinical strains are needed.

The aim of the present study was to reveal the effect of xylitol and erythritol on the growth and biofilm formation of cariogenic bacteria *S. mutans*, *S. sobrinus* and *S. wiggsiae*, also to assess the possible synergistic influence of these polyols.

## Results

### Cariogenic bacterial growth inhibition by different erythritol and xylitol concentrations

Inhibitory effect of different concentrations (15%, 7.5%, 3.75%, 1.9% and 0.9%) of polyols was tested on two clinical strains of *S. sobrinus* (*S. sobrinus* HUMB 13000, *S. sobrinus* HUMB 13104), two clinical strains of *S. mutans* (*S. mutans* HUMB 13076, *S. mutans* HUMB 13034) and *S. wiggsiae* DSM 22547 strain. Clinical strains were chosen on the basis of their origin - one adult and one child oral strain (Table 1). Individual strain data is provided in the supplementary materials **(**Supplementary Fig. 1a,b**)**. Though the optical density was measured in different time points during the experiments the detectable growth was achieved only by 24 h (data not shown) and consequently 24 h data were analysed.

**Table 1:** The list of bacterial strains used in the study.

No	Microbe	Collection number	Origin of the strain
1	*Streptococcus sobrinus*	HUMB 13000	oral cavity, child
2	*Streptococcus sobrinus*	HUMB 13087	oral cavity, child
3	*Streptococcus sobrinus*	HUMB 13104	oral cavity, adult
4	*Streptococcus sobrinus*	HUMB 13038	oral cavity, adult
5	*Streptococcus sobrinus*	HUMB 13105	oral cavity, adult
6	*Streptococcus mutans*	HUMB 13005	oral cavity, child
7	*Streptococcus mutans*	HUMB 13076	oral cavity, child
8	*Streptococcus mutans*	HUMB 13034	oral cavity, adult
9	*Streptococcus mutans*	HUMB 13033	oral cavity, adult
10	*Streptococcus mutans*	HUMB 13102	oral cavity, adult
11	*Scardovia wiggsiae*	DSM 22547	oral cavity, type strain

**Figure 1:**
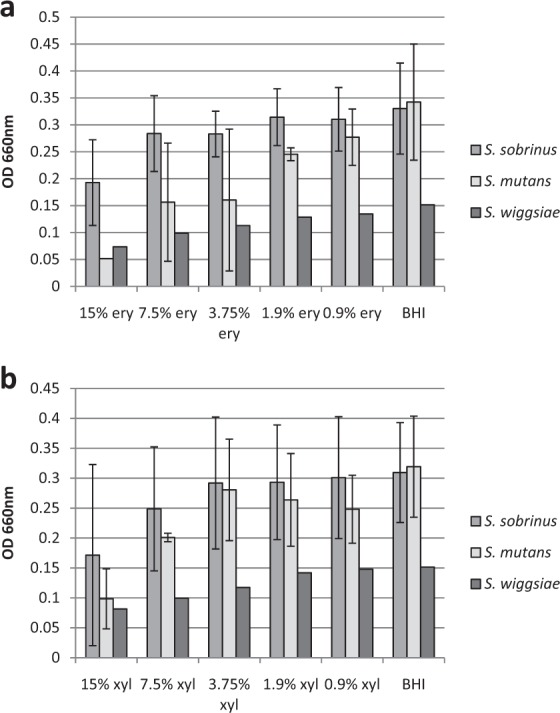
Inhibitory effect of erythritol (**a**) and xylitol (**b**) to the growth of studied two *S. sobrinus*, (*S. sobrinus* HUMB 13000, *S. sobrinus* HUMB 13104) two *S. mutans* (*S. mutans* HUMB 13076, *S. mutans* HUMB 13034) and *S. wiggsiae* DSM 22547 strain after 24 h incubation. The growth was measured spectrophotometrically by determining the optical density at 660 nm. Average of two median results from triplicate experiments and standard deviations of the studied strains in are presented.

Bacterial growth inhibition by different erythritol and xylitol concentrations was strain specific, but in average similarly high inhibitory effect (inhibition more than 40%) of both polyols at 15% concentration to all studied cariogenic bacteria (Fig. 1a,b) was found. The strains of *S. mutans* were more sensitive to polyols than two other cariogenic bacteria. The growth of S*. mutans* was inhibited up to 85% by 15% erythritol, more than 50% by 7.5% and also by 3.75% of erythritol and approx. 1/3 by 7.5% of xylitol **(**Supplementary Fig. 2a,b**)**.

### Synergistic effect of polyols on growth inhibition of cariogenic bacteria

According to the bacterial growth inhibition test results, 10% of final polyol concentration was chosen for studying synergistic effect of polyols. The polyols were used in different combinations and two concentrations of triclosan were used as controls.

**Figure 2:**
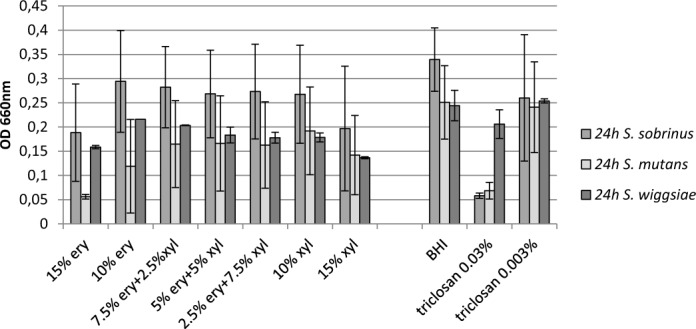
Synergistic effect of polyols and triclosan on the **growth** of 5 *S. sobrinus* (HUMB 13000, HUMB 13087, HUMB 13104, HUMB 13038, HUMB 13105), 5 *S. mutans* (HUMB 13005, HUMB 13076, HUMB 13034, HUMB 13033, HUMB 13102) and *S. wiggsiae* DSM 22547 strains after 24 h incubation. The growth was measured spectrophotometrically by determining the optical density at 660 nm. Average of two median results from triplicate experiments and standard deviations of the studied strains in are presented.

In these experiments, five clinical strains of *S. sobrinus* (*S. sobrinus* HUMB 13000, HUMB 13087, HUMB 13104, HUMB 13038, HUMB 13105) and five clinical strains of *S. mutans* (*S. mutans* HUMB 13005, HUMB 13076, HUMB 13034, HUMB 13033, HUMB 13102) and additionally *S. wiggsiae* DSM 22547 strain **(**Table 1) were used in order to get more generalized view. Inter-strain variability (shown as SD) in the growth inhibition by polyols is presented in Fig. 2. Erythritol and xylitol, in all studied combinations and individually, inhibited the growth of cariogenic bacteria compared to BHI. Both polyols in 15% of concentrations were the most effective.

Within the three combinations tested (7.5% ERY + 2.5% XYL, 5% ERY + 5% XYL and 2.5% ERY + 7.5% XYL), the effect on *S. mutans, S. sobrinus* and *S. wiggsiae* remained relatively consistent (Fig. 2). We found 10% polyol combination with excess of erythritol to be more effective against *S. mutans* and the combination with excess of xylitol more effective against *S. sobrinus* and *S. wiggsiae* (Fig. 2). Still, 15% of polyol concentration showed higher inhibitory effect on the cariogenic bacteria than any of polyol 10% combinations had). The solution of 0.03% of triclosan, served as a positive control, had high effect (growth inhibition more than 70%) against mutans streptococci but not against *S. wiggsiae* (Supplementary Fig. 3**)**. At the same time, the lower triclosan concentration (0.003%) did not have growth inhibitory effect against cariogenic bacteria.

### Inhibition of biofilm formation

**Figure 3:**
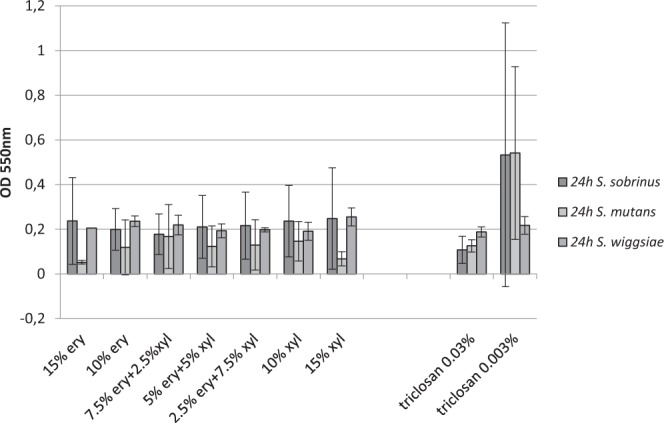
Synergistic effect of polyols and triclosan on the inhibition of **biofilm** of 5 *S. sobrinus* (HUMB 13000, HUMB 13087, HUMB 13104, HUMB 13038, HUMB 13105), 5 *S. mutans* (HUMB 13005, HUMB 13076, HUMB 13034, HUMB 13033, HUMB 13102) and *S. wiggsiae* DSM 22547 strains after 24 h incubation. The biofilm was measured spectrophotometrically by determining the optical density at 550 nm. Average of two median results from triplicate experiments and standard deviations of the studied strains in are presented.

The biofilm inhibition of cariogenic bacteria by polyols was strain specific. The results of the biofilm inhibition tests of the cariogenic bacteria are presented in Fig. 3. Solutions of 10% polyols in different combinations and 15% single polyols were equally effective against mutans streptococci (biofilm inhibition of *S. sobrinus* 57–69% and for *S. mutans* 63–85%; Supplementary Fig. 4). On the contrary, we found higher biofilm formation of *S. wiggsiae* compared to BHI in different polyol concentrations for 1–34%. Biofilm formation of *S. wiggsiae* was increased in the 10% polyol combinations for 2–15%, in 10% single polyol solutions for 1–24% and in 15% single polyol solution for 8–34%. (Fig. 3, Supplementary Fig. 4).

The control solution, 0.03% triclosan, inhibited biofilm formation of mutans streptococci (85% for *S. sobrinus* and 66% for *S. mutans*) while had no effect on *S. wiggsiae*. The 0.003% triclosan solution had moderate inhibitory effect (36%) on *S. sobrinus*, but biofilm-supporting effect on *S. mutans* (−5%) and *S. wiggsiae* (−14%).

Altogether, 10% polyol concentrations like also 15%, were effective against mutans streptococci, inhibiting their growth and biofilm formation. The growth of *S. wiggsiae* was also inhibited by polyols, but at the same time biofilm formation was increased.

## Discussion

Our experiments showed consistently growth inhibitory effect of polyols, both singularly and in combinations, on the set of clinical strains of mutans streptococci and newly detected cariogenic species *S. wiggsiae*. At the same time, the inhibition of biofilm formation by polyols was dependent on the type of organism and the polyol(s) studied. For example, inhibition of biofilm formation was clearly evident in case of mutans streptococci and mostly missing or even biofilm enhancing in case of *S. wiggsiae*.

Xylitol was one of the first sugar alcohol acknowledged by its effect against cariogenic bacteria in 1970ies. In recent years another polyol, erythritol, has been found to possess similar effect^4^. In concordance with previous studies^11^, we found that *S. mutans* was more sensitive to polyols than other studied cariogenic bacteria (*S.sobrinus* and *S. wiggsiae*). Though even very low (0.01%) polyol concentrations are able to inhibit the growth of mutans streptococci^12^, we found high polyol concentrations (15%) to be the most effective in growth inhibition.

Singularly, both erythritol and xylitol were found to be effective in the growth inhibition of the cariogenic bacteria in our study although strain-specific differences existed. Differences between individual strains of cariogenic bacteria have also been found in previous studies^4,11^. Accordingly, several clinical isolates of different hosts were used for our experiments to get more reliable results. Inhibition of biofilm formation in case of mutans streptococci was high and even more expressed than growth inhibition. No association between magnitudes of inhibition of growth and adherence was found previously^11^. At the same time, we found that although the growth inhibition of *S. wiggsiae* by polyols was significant, the biofilm formation was even increased. Söderling & Hietala-Lenkkeri^11^ presented similar results where erythritol decreased glass surface adherence of mutans streptococci, with the exception of *S. salivarius* in which case the adherence was increased.

Several compounds have been found to be synergistic with xylitol in inhibiting biofilm formation of mutans streptococci^13,14^. In our study, we explored the potential synergistic effect of xylitol and erythritol. In that regard, we used xylitol and erythritol combinations in 10% final concentration as we found it to be optimal against mutans streptococci, inhibiting their growth and biofilm formation. At the same time, all 10% polyol combinations had lower growth inhibition effect on the cariogenic bacteria than high (15%) individual polyol concentrations, but probably more difficult to achieve in oral cavity. The polyol combinations with higher proportion of erythritol were more active in growth inhibition of *S. mutans*. Similar implication about erythritol being more effective than xylitol against *S. mutans* induced dental problems has been made by de Cock *et al*.^15,16^. At the same time the combination with higher proportion of xylitol were more active in growth inhibition of *S. sobrinus* and *S. wiggsiae*. Given the emerging role of *S. wiggsiae* in advanced and initial caries^17^, this observed trade off might be relevant to take into account when choosing the use of different individual or combinations of polyols for applications. No clear synergistic effects were found for inhibition of growth and biofilm formation concerning the studied cariogenic bacteria. Overall, based on the various effects on distinct species of cariogenic bacteria by erythritol, xylitol and their combinations, personalized use of polyols may be suggested according to patient’s existing pathogens.

Triclosan is a broad-spectrum antibacterial agent widely used in hygiene especially oral care products. The solution of 0.03% of triclosan had high inhibitory effect on the growth and biofilm formation of mutans streptococci. The inhibitory effect was comparable to high polyol concentrations. At the same time, no significant effect on the growth or biofilm formation of 0.03% triclosan on *S. wiggsiae* was found. The lower, 0.003% triclosan concentration showed positive inhibition effect on *S. sobrinus* biofilm formation, but slight biofilm enhancing effect on *S. mutans* and *S. wiggsiae*. Although antibacterial effect of triclosan on *S. sobrinus* and *S. mutans* is widely acknowledged^18,19^ its effect on biofilm formation is not fully investigated. Sub-inhibitory triclosan concentrations have shown to enhance the biofilm formation of *S. mutans*^18^ but to our best knowledge no respective results on *S. sobrinus* and *S. wiggsiae* have been published before.

Limitation of our study include moderate number of tested strains and lack of type strains in case of mutans streptococci. At the same time, our study included two different polyols and three different species of cariogenic bacteria, including also novel species *S. wiggsiae*.

## Conclusions

Both erythritol and xylitol as well as their combinations inhibit the growth of clinical strains of mutans streptococci and *S. wiggsiae*, newly recognized cariogenic bacterium. Biofilm formation of mutans streptococci is also strongly inhibited. When applying polyols (singularly or in combinations) in caries prophylaxis, it is relevant to consider the profile of pathogens in a particular patient and thus optimize the choice of polyols based on this profile.

## Materials and Methods

### Chemicals and strains

Erythritol (Cargill R&D Centre Europe, Vilvoorde, Belgium) and xylitol (≥99%, Sigma-Aldrich Co, St. Louis, USA) were tested.

Altogether 11 cariogenic bacteria (5 *Streptococcus sobrinus*, 5 *Streptococcus mutans* clinical isolates and 1 *Scardovia wiggsiae* DSM 22547 type strain**;** Table 1) were included into the study. The clinical isolates were acquired from the Human Microbiota Biobank of Tartu (HUMB; http://www.eemb.ut.ee/eng/).

### Bacterial growth inhibition by different polyol concentrations

The effect of polyols was studied using slightly modified method previously described by Mäkinen, *et al*.^2,4^. Briefly, the tested substances were sterilized by filtration at desired concentration and added to the brain-heart infusion (BHI, Oxoid Limited, Basingstoke, Hampshire, UK) medium (sterilization by autoclaving at 121 °C for 15 min). The bacteria were incubated in aerobic conditions at 37 °C for 24 hours on blood agar (Oxoid Limited, Basingstoke, Hampshire, UK) (*S. mutans, S. sobrinus*) or in anaerobic conditions with gases: 90%N, 5%CO_2_, 5% H_2_ (Whitley Anaerobic Workstations A35, Don Whitley Scientific Limited, Bingley, West Yorkshire, UK) at 37 °C for 24 hours on fastidious anaerobe agar (*S. wiggsiae*) (LAB090, Lab M Limited, Heywood, Lancashire, UK). The microtiter plate wells (CELLSTAR, 96 well polystyrene suspension culture microplates, F-bottom, Greiner Bio-One GmbH, Kremsmünster, Austria) were inoculated with equal amounts (200 µl) of bacterial (the final test-concentration of 10^5^ CFU/ml) and polyol solution. The bacterial cells were grown in aerobic or anaerobic conditions at 37 °C on the microtiter plate, monitoring the growth up to the late exponential growth phase. The density of bacterial growth was detected spectrophotometrically in absorbance microplate reader (Sunrise, Tecan Group Ltd., Männedorf, Switzerland) at 660 nm. The time points for measuring were 0, 1, 2, 4, 6 and 24 hours.

The cultivations were carried out with all organisms, using the polyol concentrations as indicated in Table 2 as well as negative (BHI) and positive (triclosane) controls. All bacterial strains were tested in 3 repeats for 2 times.

**Table 2:** Polyol and triclosan concentrations used in the study.

	Inhibition study		Synergistic study		Biofilm study	
	Polyol/triclosan concentration (%)					
Cariogenic pathogens	ERY	XYL	Polyol	Triclosan	Polyol	Triclosan
*S. sobrinus, S mutans, S. wiggsiae*	15	15	15% ERY	0.03	15% ERY	0.03
	7.5	7.5	10% ERY	0.003	10% ERY	0.003
	3.75	3.75	7.5% ERY + 2.5%XYL		7.5% ERY + 2.5%XYL	
	1.9	1.9	5% ERY + 5% XYL		5% ERY + 5% XYL	
	0.9	0.9	2.5% ERY + 7.5% XYL		2.5% ERY + 7.5% XYL	
			10% XYL		10% XYL	
			15% XYL		15% XYL	

ERY erythritol, XYL xylitol.

The tested polyol concentrations (weight/volume) included 15%, 7.5%, 3.75%, 1.9%, and 0.9% (Table 2). The growth inhibition percentages were calculated as ratios between average tests and BHI values.

### Synergistic effect of polyols on growth inhibition of cariogenic bacteria *in vitro*

After defining the inhibitory effect of erythritol and xylitol in total of 5 concentrations for *S. wiggsiae* and two strains of *S. sobrinus* and *S. mutans*, the most optimal summary polyol concentration was defined and used in further studies in different proportions (Table 2).

In addition, growth inhibition capacity of triclosan as positive control was tested in two concentrations (0.03% and 0.003%)^18^. The growth inhibition percentages were calculated as ratios between average tests and BHI values.

### Inhibition of biofilm formation testing

Inhibition of bacterial biofilm production by polyols in BHI solution was measured using a semi-quantitative adherence assay on 96-well microtiter plates (CELLSTAR, 96 well polystyrene suspension culture microplates, F-bottom)^20,21^. The bacteria were cultivated at 37 °C for 24 h on blood agar (*S. mutans, S. sobrinus*) or fastidious anaerobe agar (*S. wiggsiae*). The microtiter plate wells were inoculated with equal amounts (200 µl) of bacterial (the final test-concentration of 10^5^ CFU/ml) and polyol solution. The plates were covered and incubated aerobically or anaerobically (90%N, 5%CO_2_, 5% H_2_) at 37 °C for 24 h. At the end of incubation, the liquid in the wells was poured out and each well was washed three times with 0.25 ml of sterile phosphate buffered saline (PBS).

Plates were stained for 10 min with 0.1 ml of 0.04% crystal violet per well. The wells were washed four times with distilled water to remove the unbound crystal violet dye and dried for 2 h at 37 °C. After adding 0.1 mL of 95% (v/v) ethanol to each well, optical density (OD) in absorbance microplate reader at 550 nm was measured with the preceding shaking of 5 min with the amplitude of 5 mm to release the stain from the biofilms.

Inhibition of biofilm of cariogenic bacteria was tested using different polyol concentrations alone and in combinations (XYL 15%, XYL 10%, XYL 7.5% + ERY 2.5%, XYL 5% + ERY 5%, XYL 2.5% + ERY 7.5%, ERY 10%, ERY 15%) (Table 2). Biofilm inhibition capacity of triclosan as positive control was tested in two concentrations (0.03% and 0.003%)^18^. All bacterial strains were tested in 3 repeats for 2 times.

The biofilm inhibition percentages were calculated as ratios between tests averages and BHI values.

### Statistical analysis

All experiments were performed in triplicate and repeated twice. Each value represents the average of two median results from triplicate experiments.

## Supplementary information

Supplementary Information.

## Data Availability

The datasets generated during and/or analysed during the current study are available from the corresponding author on reasonable request.
