# Supplementary material for: Exploration of singular and synergistic effect of xylitol and erythritol on causative agents of dental caries
Source: Sci Rep. 2020 Apr 14;10:6297. doi: 10.1038/s41598-020-63153-x (PMC7156733; doi:10.1038/s41598-020-63153-x)
Supplement: Supplementary file 1 — Supplementary Information. [file 41598_2020_63153_MOESM1_ESM.pdf]

Supplementary information

Exploration of singular and synergistic effect of xylitol and erythritol on causative agents of dental caries

Siiri Kõljalg*[1], Imbi Smidt[1], Anirikh Chakrabarti[2], Douwina Bosscher[2], Reet Mändar[1]

[1] Department of Microbiology, Institute of Biomedicine and Translational Medicine, University of Tartu, Tartu, Estonia

[2] Cargill R&D Centre Europe, Vilvoorde, Belgium

* corresponding author

Siiri Kõljalg siiri.koljalg@ut.ee

Imbi Smidt imbi.smidt@ut.ee

Anirikh Chakrabarti Anirikh_Chakrabarti@cargill.com

Douwina Bosscher Douwina_Bosscher@cargill.com

Reet Mändar reet.mandar@ut.ee

Fig 1a

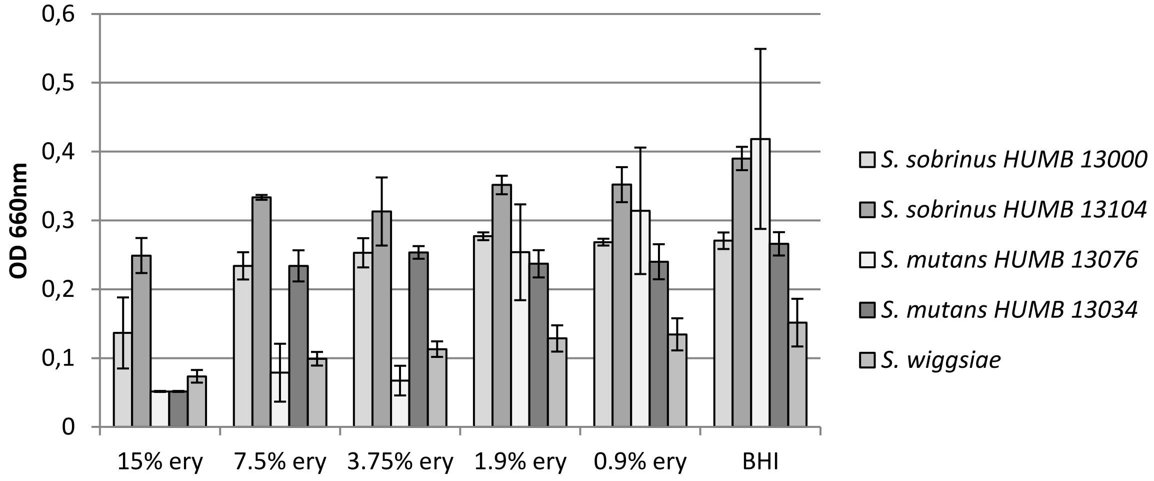

Fig 1b

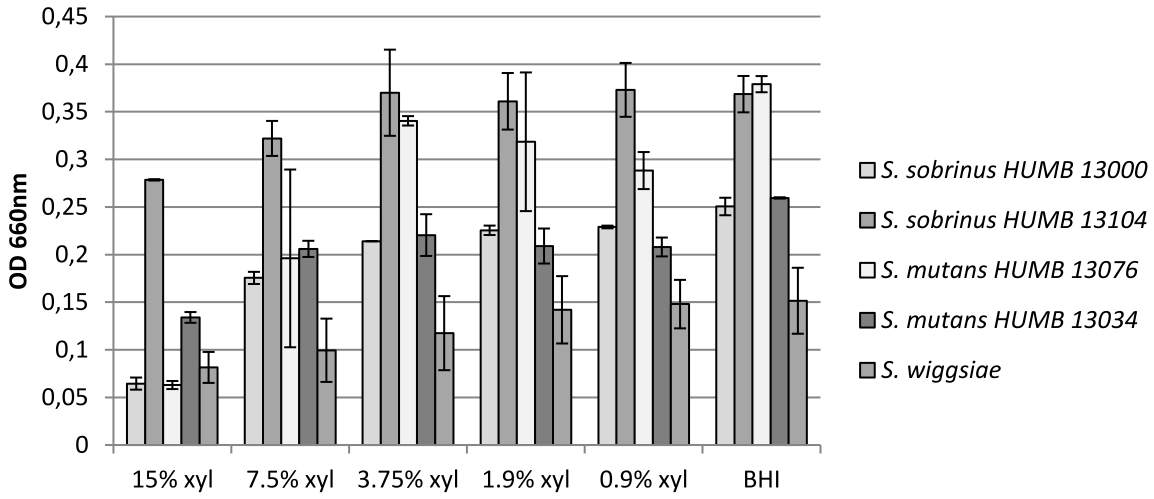

Supplementary Figure 1. Inhibitory effect of erythritol (a) and xylitol (b) to the growth of studied two *S. sobrinus*, (*S. sobrinus* HUMB 13000, *S. sobrinus* HUMB 13104) two *S. mutans* (*S. mutans* HUMB 13076, *S. mutans* HUMB 13034) and *S. wiggsiae* DSM 22547 strain after 24 h incubation. The growth was measured spectrophotometrically by determining the optical density at 660 nm. Average of two median results from triplicate experiments and standard deviations are presented.

Fig 2a

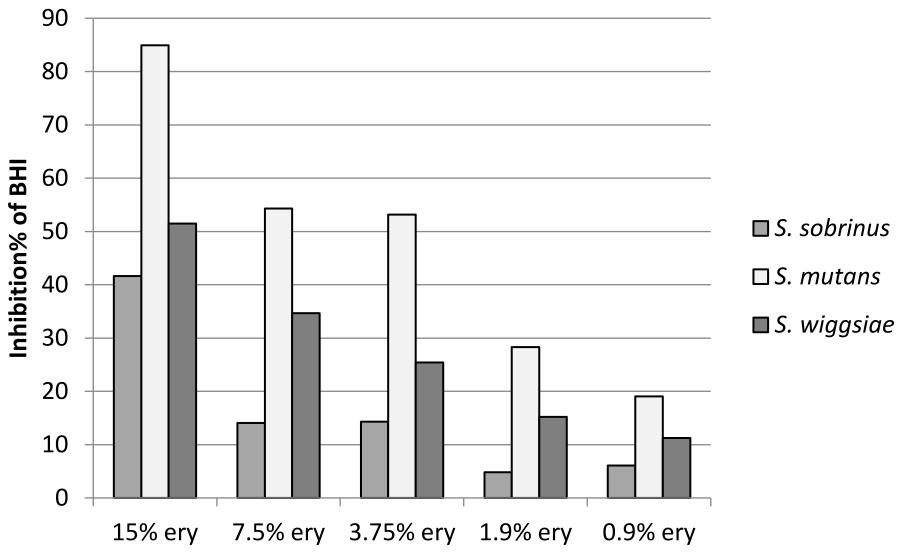

Fig 2b

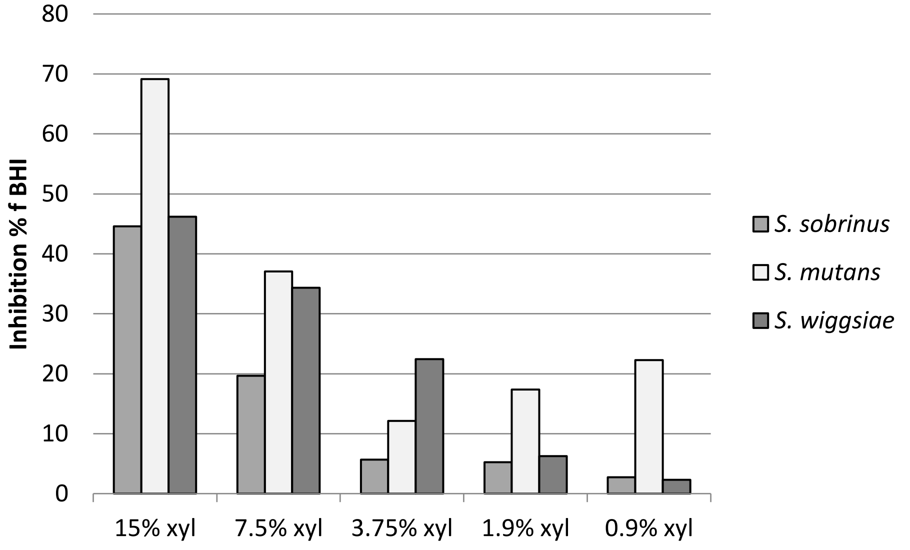

Supplementary Figure 2. Inhibitory effect (%) erythritol (a) and xylitol (b) to the growth of studied two *S. sobrinus*, (*S. sobrinus* HUMB 13000, *S. sobrinus* HUMB 13104) two *S. mutans* (*S. mutans* HUMB 13076, *S. mutans* HUMB 13034) and *S. wiggsiae* DSM 22547 strain after 24 h incubation. The growth was measured spectrophotometrically by determining the optical density at 660 nm. Average of two median results from triplicate experiments was calculated and inhibition compared to the growth in BHI is presented

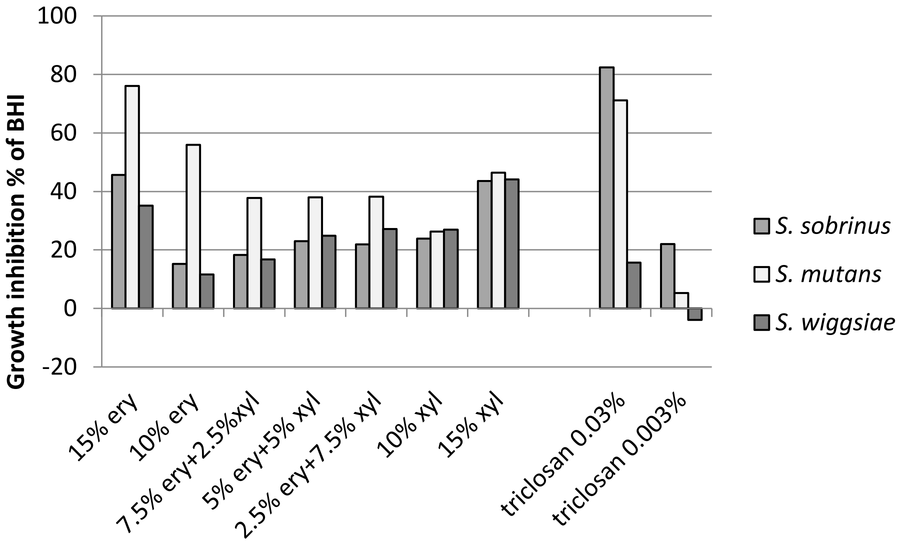

Supplementary Figure 3. Average growth inhibition of polyol combinations and triclosan (% of BHI) 5 S. sobrinus (HUMB 13000, HUMB 13087, HUMB 13104, HUMB 13038, HUMB 13105), 5 S. mutans (HUMB 13005, HUMB 13076, HUMB 13034, HUMB 13033, HUMB 13102) and S. wiggsiae DSM:22547 strains after 24 The growth was measured spectrophotometrically by determining the optical density at 660 nm. Average of two median results from triplicate are presented as percentage of BHI

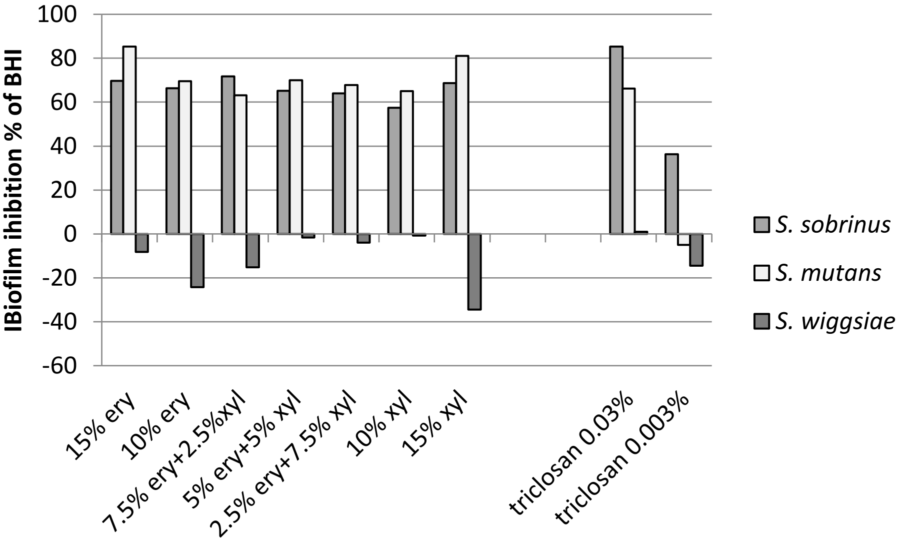

Supplementary Figure 4. Average biofilm inhibition of polyol combinations and triclosan (% of BHI) 5 S. sobrinus (HUMB 13000, HUMB 13087, HUMB 13104, HUMB 13038, HUMB 13105), 5 S. mutans (HUMB 13005, HUMB 13076, HUMB 13034, HUMB 13033, HUMB 13102) and S. wiggsiae DSM:22547 strains after 24 The biofilm was measured spectrophotometrically by determining the optical density at 550 nm. Average of two median results from triplicate are presented as percentage of BHI
